# Off-Grid DOA Estimation Using Alternating Block Coordinate Descent in Compressed Sensing

**DOI:** 10.3390/s150921099

**Published:** 2015-08-27

**Authors:** Weijian Si, Xinggen Qu, Zhiyu Qu

**Affiliations:** Department of Information and Communication Engineering, Harbin Engineering University, Harbin 150001, China; E-Mails: swj0418@263.net (W.S.); quxinggen@126.com (X.Q.)

**Keywords:** alternating block coordinate descent, block selection criterion, block sparse source, compressed sensing, off-grid direction of arrival (DOA) estimation

## Abstract

This paper presents a novel off-grid direction of arrival (DOA) estimation method to achieve the superior performance in compressed sensing (CS), in which DOA estimation problem is cast as a sparse reconstruction. By minimizing the mixed *k*-*l* norm, the proposed method can reconstruct the sparse source and estimate grid error caused by mismatch. An iterative process that minimizes the mixed *k*-*l* norm alternately over two sparse vectors is employed so that the nonconvex problem is solved by alternating convex optimization. In order to yield the better reconstruction properties, the block sparse source is exploited for off-grid DOA estimation. A block selection criterion is engaged to reduce the computational complexity. In addition, the proposed method is proved to have the global convergence. Simulation results show that the proposed method has the superior performance in comparisons to existing methods.

## 1. Introduction

Direction of arrival (DOA) estimation has played an important role in many fields, such as radar, medical imaging and array signal processing [1,2]. In the last decades, many classical methods have been developed, among which multiple signal classification (MUSIC) [3] and estimation of signal parameter via rotational invariance technique. (ESPRIT) [4] are the most popular and have high resolution for DOA estimation. However, these methods are very sensitive to the number of snapshots, signal to noise ratio (SNR) and the correlation between sources. Small number of snapshots, low SNR and high correlation or coherent sources can all make the performance of these methods degrade severely. More recently, the emerging field of compressed sensing (CS) [5,6] has attracted enormous attention and it can reconstruct the sparse source using nonadaptive linear projection measurement obtained by the measurement matrix that satisfies the restricted isometry property (RIP) [7,8]. Support denotes the set that contains the indices of the nonzero elements in the sparse source. Once the support is determined, the sparse source can be reconstructed.

Due to the fact that sources are intrinsically sparse in the spatial domain, the DOA estimation problem can be regarded as a sparse reconstruction in the framework of CS. The CS-based estimation methods have much better estimation performance than conventional estimation methods. Malioutov *et al.* [9] firstly adopted the sparse signal reconstruction (SSR) perspective for DOA estimation and utilized the singular value decomposition (SVD) of the data matrix to propose $l_{1}$-SVD method. In [10], CS-MUSIC was proposed by revisiting the link between CS and MUSIC. This method identifies the parts of support using CS, after which the remaining parts are estimated by a novel generalized MUSIC criterion. Xu *et al*. [11] utilized the Capon spectrum to design a weighted $l_{1}$-norm penalty in order to further enforce the sparsity and approximate the original $l_{0}$-norm for DOA estimation. Wei *et al*. [12] proposed a modified greedy block coordinate descent (R-GBCD) method and the corresponding version with weight refinement (R-GBCD+) to improve the estimation performance.

The key to guarantee the performance of conventional CS-based estimation methods is that all true DOAs are exactly located on the grid. However, when true DOAs are not on the grid set, the performance may severely degrade due to the grid error caused by mismatch, which is defined as the distance from the true direction to the nearest grid. In order to address this issue, Zhu *et al.* [13] proposed the sparse total least square (STIS) to perform the off-grid DOA estimation, in which perturbations of the model are assume to be Gaussian. In [14], Yang *et al*. introduced the Bayesian theory in off-grid DOA estimation and proposed an off-grid sparse Bayesian inference based on the singular value decomposition (OGSBI-SVD). Liang *et al*. [15] proposed an off-grid synchronous approach based on distributed compressed sensing to obtain larger array aperture. Zhang *et al*. [16] formulated a novel model based on the sampling covariance matrix and solved the off-grid DOA estimation problem by the block sparse Bayesian method even if the number of sources are unknown.

In this paper, a novel alternating block coordinate descent method called ABCD is proposed for off-grid DOA estimation in CS. The proposed method solves the mixed *k*-*l* norm minimization problem to reconstruct the sparse source and estimate the grid error. Since joint estimation will lead to a nonconvex optimization problem, the proposed method adopts an iterative process that minimizes the mixed *k*-*l* norm alternately over two sparse vectors. Instead of conventional sparse source, the block sparse source is exploited to achieve better reconstruction properties. The block is updated by the proposed block selection criterion, which can improve efficiency of the proposed method. In addition, we give a detailed derivation process of proving the global convergence of the proposed method. Simulation results illustrate the superior performance of the proposed method as compared with existing methods.

The rest of the paper is organized as follows. An off-grid DOA estimation model is formulated in Section 2 and Section 3 introduces the proposed method in detail. The global convergence of the proposed method is proved in Section 4 and Section 5 shows the performance of the proposed method. Conclusions are provided in Section 6.

## 2. Problem Formulation

Consider *K* far-field narrowband sources $s_{1}(t),s_{2}(t),\cdots ,s_{K}(t)$ impinging on the uniform linear array (ULA) consisting of *M* omnidirectional sensors with inter-sensor spacing $\overset{⌢}{d}$. Assume that each source $s_{k}(t)$ is located at different direction $\theta_{k}$ with the power ${\text{σ}}_{k}$, k=1,2,⋯,K. At time instant *t*, the received source by ULA can be expressed as

(1) $$x(t)=\sum_{k=1}^{K}a(\theta_{k})s_{k}(t)+n(t)\;\;t=1,2\cdots T$$

where $a(\theta_{k})\in {\mathbb{C}}^{M\times 1}$ and $n(t)\in {\mathbb{C}}^{M\times 1}$ denote the steering vector and noise vector, respectively. Since the first point of ULA is set as the origin of sensor array, the *m*th element of $a(\theta_{k})$ is written as $e^{-j(\text{2π}/\text{λ})\hat{d}(m-1)\cos\theta_{k}}$ with the wavelength of source λ.

To incorporate the CS theory with the DOA estimation, the entire angular space is divided into a fine grid $\tilde{\theta }={\left[{\tilde{\theta }}_{1},{\tilde{\theta }}_{2}\cdots {\tilde{\theta }}_{L}\right]}^{T}$, where *L*(L≫K) denotes the number of the grid and ${\left[\cdot \right]}^{T}$ denotes the transpose. Due to the fact that true directions $\theta ={\left[\theta_{1},\theta_{2}\cdots \theta_{K}\right]}^{T}$ are random in the entire angular space, $\theta_{k}$ for some k∈{1,2⋯K} are likely to be not on the grid set. To reduce the grid error caused by mismatch, we formulate the off-grid model, which has a close relationship with the on-grid model. Let $\tilde{\theta }$ satisfy the uniform distribution so that the grid interval $\text{τ}={\tilde{\theta }}_{k+1}-{\tilde{\theta }}_{k}\propto L^{-1}$. Without loss of generality, by assuming $\theta_{k}\notin \{{\tilde{\theta }}_{1},{\tilde{\theta }}_{2}\cdots {\tilde{\theta }}_{L}\}$ and that ${\tilde{\theta }}_{l_{k}}\in \{{\tilde{\theta }}_{1},{\tilde{\theta }}_{2}\cdots {\tilde{\theta }}_{L}\}$ is the nearest grid to $\theta_{k}$, the steering vector $a(\theta_{k})$ can be approximated as

(2) $$a(\theta_{k})\approx a({\tilde{\theta }}_{l_{k}})+b({\tilde{\theta }}_{l_{k}})(\theta_{k}-{\tilde{\theta }}_{l_{k}})$$

where ${\text{ε}}_{l}=\theta_{k}-{\tilde{\theta }}_{l_{k}}\in [-\frac{\text{τ}}{\text{2}},\frac{\text{τ}}{\text{2}}]$ is the grid error and $b({\tilde{\theta }}_{l_{k}})$ is the partial derivative of $a({\tilde{\theta }}_{l_{k}})$ with respective to ${\tilde{\theta }}_{l_{k}}$. Then, if ${\tilde{\theta }}_{l_{1}},{\tilde{\theta }}_{l_{2}}\cdots {\tilde{\theta }}_{l_{K}}$ are respectively nearest grids to the true directions $\theta_{1},\theta_{2}\cdots \theta_{K}$, we have

(3) $$\left\{ \begin{array}{ccc} g_{l}(t)=s_{l_{k}}(t),{\text{ε}}_{l}=\theta_{k}-{\tilde{\theta }}_{l_{k}} & ; & l=l_{k}(k=1,2,\cdots K)\\ g_{l}(t)={\text{ε}}_{l}=0 & ; & elsewhere \end{array}\right.$$

Then, by imposing the approximation error on the noise, the received source x(t) can be rewritten as the following sparse form

(4) $$x(t)=[A+Bdiag(\text{ε})]g(t)+\overset{⌢}{n}(t)\;\;t=1,2,\cdots T$$

where $A=[a({\tilde{\theta }}_{1}),a({\tilde{\theta }}_{2}),\cdots ,a({\tilde{\theta }}_{L})]$ is the M×L array manifold matrix corresponding to all potential directions, which is defined as an overcomplete dictionary in CS, and $B=[b({\tilde{\theta }}_{1}),b({\tilde{\theta }}_{2}),\cdots ,b({\tilde{\theta }}_{L})]$. In addition, the matrix $\overset{⌢}{N}=[\overset{⌢}{n}(1),\overset{⌢}{n}(2),\cdots ,\overset{⌢}{n}(T)]$ is the noise matrix and diag(ε) is a diagonal matrix with ε being the diagonal elements. Since $g(t)={\left[g_{1}(t),g_{2}(t),\cdots ,g_{L}(t)\right]}^{T}$ has *K* nonzero elements in *L* elements, it is a *K*-sparse vector, where *K* is referred to as sparsity. More specifically, $\text{ε}={\left[{\text{ε}}_{1},{\text{ε}}_{2},\cdots ,{\text{ε}}_{L}\right]}^{T}$ is also a *K*-sparse vector and has the same support as g(t). It is evident that the on-grid sparse model is a special case by simply setting ε=0 in Equation (4). Since ${\left\{g(t)\right\}}_{t=1}^{T}$ are jointly *K*-sparse, the matrix $G=[g(1),g(2),\cdots ,g(T)]\in {\mathbb{C}}^{L\times T}$ has *K* nonzero rows and is called row *K*-sparse.

To solve the off-grid DOA estimation problem, we need to jointly estimate the support of sparse sources, g(t),t=1,2,⋯,T, and grid error ε from the matrix ***Y*** which is given by

(5) $$Y=[y(1)\;y(2)\cdots y(T)]=\text{Φ}AG+\text{Φ}Βdiag(\text{ε})G+\tilde{N}$$

where $\tilde{N}=\text{Φ}\overset{⌢}{N}$ is the N×T noise matrix, Φ is the N×M measurement matrix with N<M and *N* is the number of nonadaptive linear projection measurement.

## 3. Off-DOA Estimation

In this section, an alternating block coordinate descent (ABCD) method and a block selection criterion are elaborated in the CS scenario. The proposed method not only has the advantages of conventional BCD [17] strategy, but also uses an iterative process that minimizes the mixed *k*-*l* norm alternately over two sparse vectors. Note that due to solving the minimization alternately, a tractable convex problem is obtained and the global convergence of ABCD can be easily determined, which is proved in the next section.

By applying the central limit theorem, the components $\overset{⌢}{n}(t)$, t=1,2,⋯,T, of $\overset{⌢}{N}$ are independently white Gaussian noise with zero mean and covariance ${\text{σ}}^{2}I_{M}$, where $I_{M}$ denotes an M×M identity matrix. Thus, the covariance matrix of Y with the size M×M is expressed as

(6) $$R_{Y}=E[y(t)y^{H}(t)]=\text{Φ}AR_{G}A^{H}{\text{Φ}}^{H}+\text{Φ}Βdiag(\text{ε})R_{G}diag^{H}(\text{ε}){Β}^{H}{\text{Φ}}^{H}+{\text{σ}}^{2}{\text{ΦΦ}}^{H}$$

where $R_{G}=E[s(t)s^{H}(t)]$ is a L×L covariance matrix of the sparse source and ${\left(\cdot \right)}^{H}$ denotes the conjugate transpose. Since all potential directions are one to one corresponding to the powers ${\text{σ}}_{1}^{2},{\text{σ}}_{2}^{2},\cdots ,{\text{σ}}_{L}^{2}$ and we are interested in estimating DOAs, $R_{G}$ can be reduced to a diagonal matrix $R_{G}=diag(p)$, where $p={\left[{\text{σ}}_{1}^{2},{\text{σ}}_{2}^{2},\cdots ,{\text{σ}}_{L}^{2}\right]}^{T}$ is a *K*-sparse vector. Then, by denoting $\hat{Α}=\text{Φ}Α=[\hat{a}({\tilde{\theta }}_{1}),\hat{a}({\tilde{\theta }}_{2}),\cdots ,\hat{a}({\tilde{\theta }}_{L})]$ and $\hat{Β}=\text{Φ}Β=[\hat{b}({\tilde{\theta }}_{1}),\hat{b}({\tilde{\theta }}_{2}),\cdots ,\hat{b}({\tilde{\theta }}_{L})]$, (6) can be further rewritten as

(7) $$R_{Y}=\sum_{l=1}^{L}\hat{a}({\tilde{\theta }}_{l}){\hat{a}}^{H}({\tilde{\theta }}_{l}){\text{σ}}_{l}^{2}+\sum_{l=1}^{L}\hat{b}({\tilde{\theta }}_{l}){\hat{b}}^{H}({\tilde{\theta }}_{l}){\text{σ}}_{l}^{2}{\text{ε}}_{l}^{2}+{\text{σ}}^{2}{\text{ΦΦ}}^{H}$$

Due to the vector form of $R_{Y}$ in Equation (7), the following measurement vector is given by

(8) $$z=vec(R_{Y})=Cp+Dq+{\text{σ}}^{2}{\text{ΦΦ}}^{H}$$

with $C=[{\hat{a}}^{H}({\tilde{\theta }}_{1})⊗\hat{a}({\tilde{\theta }}_{1}),\;{\hat{a}}^{H}({\tilde{\theta }}_{2})⊗\hat{a}({\tilde{\theta }}_{2}),\cdots ,\;{\hat{a}}^{H}({\tilde{\theta }}_{L})⊗\hat{a}({\tilde{\theta }}_{L})]$ and $D=[{\hat{b}}^{H}({\tilde{\theta }}_{1})⊗\hat{b}({\tilde{\theta }}_{1}),\;{\hat{b}}^{H}({\tilde{\theta }}_{2})⊗\hat{b}({\tilde{\theta }}_{2}),\cdots ,{\hat{b}}^{H}({\tilde{\theta }}_{L})⊗\hat{b}({\tilde{\theta }}_{L})]$, where ⊗ and vec(·) denote the Kronecker product and the stack operation by placing the columns of a matrix on the top of one another in order, respectively. Moreover, $q={\left[{\text{σ}}_{1}^{2}{\text{ε}}_{1}^{2},{\text{σ}}_{2}^{2}{\text{ε}}_{2}^{2},\cdots ,{\text{σ}}_{L}^{2}{\text{ε}}_{L}^{2}\right]}^{T}$ is also a *K*-sparse vector, which has the same support as p. In the conventional sparse source, the nonzero elements of the sparse vector p or q can appear anywhere in the vector. However, in this paper, our goal is to explicitly take block sparse source into account, *i.e.*, the nonzero elements of the sparse vector p or q tend to cluster in blocks. The motivations to exploit block sparse source are the following two main reasons. As can be seen in [18], the first reason is that block sparse source has been applied in many applications, such as unions of subspaces and multiband sources [19]. Secondly, block sparse source has better reconstruction properties than sparse source in the conventional sense, which is proved in [20]. To exploit block sparse source, denote $p[h]={\left[{\text{σ}}_{d(h-1)+1}^{2},{\text{σ}}_{d(h-1)+2}^{2},\cdots ,{\text{σ}}_{dh}^{2}\right]}^{T}$ and $q[h]={\left[{\text{σ}}_{d(h-1)+1}^{2}{\text{ε}}_{d(h-1)+1}^{2},{\text{σ}}_{d(h-1)+2}^{2}{\text{ε}}_{d(h-1)+2}^{2},\cdots ,{\text{σ}}_{dh}^{2}{\text{ε}}_{dh}^{2}\right]}^{T}$ as the *h*th blocks of p and q with the length *d*, respectively, so that we have

(9) $$p={\left[p^{T}[1],p^{T}[2],\cdots ,p^{T}[H]\right]}^{T}$$

(10) $$q={\left[q^{T}[1],q^{T}[2],\cdots ,q^{T}[H]\right]}^{T}$$

where L=dH. Following the similar manner, the matrices C and D can be respectively viewed as a concatenation of block matrices C[h] and D[h] of the size $N^{2}\times d$, *i.e.*,

(11) C=[C[1],C[2],⋯,C[H]]

(12) D=[D[1],D[2],⋯,D[H]]

where $C[h]=[{\hat{a}}^{H}({\tilde{\theta }}_{d(h-1)+1})⊗\hat{a}({\tilde{\theta }}_{d(h-1)+1}),{\hat{a}}^{H}({\tilde{\theta }}_{d(h-1)+2})⊗\hat{a}({\tilde{\theta }}_{d(h-1)+2}),\cdots ,{\hat{a}}^{H}({\tilde{\theta }}_{dh})⊗\hat{a}({\tilde{\theta }}_{dh})]$ and $D[h]=[{\hat{b}}^{H}({\tilde{\theta }}_{d(h-1)+1})⊗\hat{b}({\tilde{\theta }}_{d(h-1)+1}),{\hat{b}}^{H}({\tilde{\theta }}_{d(h-1)+2})⊗\hat{b}({\tilde{\theta }}_{d(h-1)+2}),\cdots ,{\hat{b}}^{H}({\tilde{\theta }}_{dh})⊗\hat{b}({\tilde{\theta }}_{dh})]$. Obviously, the conventional sparse source is a special case of block sparse source by simply setting d=1. If ${\left\|p[h]\right\|}_{2},h=1,2,\cdots ,H,$ has at most *K* nonzero elements, the vector p is referred to as block *K*-sparse, where ${\left\|\cdot \right\|}_{2}$ denotes the Euclidean norm for vectors. In contrast to conventional sparsity, *K* is called block-sparsity.

Since p has the same support as q, p and q are jointly sparse. Thus, a mixed *k*-*l* norm minimization problem [21] is utilized to jointly reconstruct p and q. Given a L×1 block sparse vector p, the mixed *k*-*l* norm of p is defined as

(13) $${\left\|p\right\|}_{k,l}^{l}=\sum_{h=1}^{H}{\left({\left\|p[h]\right\|}_{k}\right)}^{l}$$

Combining the definition given by Equation (13), this mixed *k*-*l* norm minimization problem is formulated as

(14) $$\underset{p,q}{\min}({\left\|p\right\|}_{k,l}^{l}+\text{β}{\left\|q\right\|}_{k,l}^{l})\;\;\;s.t.z=Cp+Dq+{\text{σ}}^{\text{2}}{\text{ΦΦ}}^{H}$$

where 0≤l≤1. It is worth mentioning that an important class of methods for solving the constrained optimization problem is to form the auxiliary function. By introducing the Lagrange multiplier method, the Lagrange function with respect to Equation (14) is given by

(15) $$\underset{p,q}{\min}\frac{1}{2}{\left\|z-Cp-Dq\right\|}_{2}^{2}+{\text{β}}_{p}\sum_{h=1}^{H}{\left({\left\|p[h]\right\|}_{k}\right)}^{l}+{\text{β}}_{q}\sum_{h=1}^{H}{\left({\left\|q[h]\right\|}_{k}\right)}^{l}$$

where ${\text{β}}_{p}$ and ${\text{β}}_{q}$ are regularized parameters. As can be seen in Equation (15), the minimization problem with respect to p and q is nonconvex so as to make DOA estimation intractable. But note that if we fix one of two sparse vectors, that is, either p or q, the minimization problem in Equation (15) turns out to be convex with respect to the other sparse vector alone. Thus, p and q can be reconstructed by alternately solving the following two minimization problems under the condition that the other sparse vector is fixed.

(16) $$\underset{p}{\min}\{\frac{1}{2}{\left\|z-Cp-Dq\right\|}_{2}^{2}+{\text{β}}_{p}\sum_{h=1}^{H}{\left({\left\|p[h]\right\|}_{k}\right)}^{l}\}$$

(17) $$\underset{q}{\min}\{\frac{1}{2}{\left\|z-Cp-Dq\right\|}_{2}^{2}+{\text{β}}_{q}\sum_{h=1}^{H}{\left({\left\|q[h]\right\|}_{k}\right)}^{l}\}$$

It is clear that Equations (16) and (17) have the same structure and can be solved in a similar manner. In the following, we only need to find the optimal solution for Equation (16). Then, the minimization problem in Equation (17) can be handled in the same way.

The objective function in Equation (16) can be expressed as

(18) F(p)=f(p)+v(p)

where $f(p)=\frac{1}{2}{\left\|z-Cp-Dq\right\|}_{2}^{2}$ and $v(p)={\text{β}}_{p}\sum_{h=1}^{H}{\left({\left\|p[h]\right\|}_{k}\right)}^{l}$. Assume that $p^{\left(k\right)}$ is obtained at the *k*th iteration. Then, by the quadratic approximation of f(p) at the fixed point $p^{\left(k\right)}$, F(p) can be approximated as

(19) $$F(p)\approx f(p^{\left(k\right)})+{\left(p-p^{\left(k\right)}\right)}^{H}\nabla f(p^{\left(k\right)})+\frac{\sum_{h=1}^{H}{\left\|p[h]-p^{\left(k\right)}[h]\right\|}_{2}^{2}}{2\Sigma }+v(p)$$

where $\nabla f(p^{\left(k\right)})=C^{H}\cdot [\sum_{h=1}^{H}(C[h]p^{\left(k\right)}[h]+D[h]q[h])-z]$ is the partial derivation of $f(p^{\left(k\right)})$ with respect to $p^{\left(k\right)}$. Σ is set to be $\Sigma =\frac{{\left\|\Delta \right\|}_{1}}{L}$, where ${\left\|\cdot \right\|}_{1}$ denotes one norm for vectors, $\Delta ={\left[\Delta_{1},\Delta_{2},\cdots ,\Delta_{L}\right]}^{T}$ is a L×1 vector and $\Delta_{l}=\frac{1}{{\left\|{\hat{a}}^{H}({\tilde{\theta }}_{l})⊗\hat{a}({\tilde{\theta }}_{l})\right\|}_{2}^{2}}$, l=1,2,⋯,L. Moreover, Δ can be shown to be a block vector, *i.e.*,

(20) $$\Delta ={\left[\Delta^{T}[1],\Delta^{T}[2],\cdots ,\Delta^{T}[H]\right]}^{T}$$

where Δ[h] is the *h*th block of Δ with the length *d*. For the convenience of analysis, denote $J^{\left(k\right)}=diag(\Delta )\nabla f(p^{\left(k\right)})$, and we can also represent $J^{\left(k\right)}$ as a block vector, *i.e.*,

(21) $$J^{\left(k\right)}=diag(\Delta )C^{H}\cdot [\sum_{h=1}^{H}(C[h]p^{\left(k\right)}[h]+D[h]q[h])-z]={\left[{\left(J^{\left(k\right)}[1]\right)}^{T},{\left(J^{\left(k\right)}[2]\right)}^{T},\cdots ,{\left(J^{\left(k\right)}[H]\right)}^{T}\right]}^{T}$$

where $J^{\left(k\right)}[h]$ is the *h*th block of $J^{\left(k\right)}$ with the length *d*. At the *k*th iteration, $p^{\left(k\right)}$ is updated by minimizing F(p) in Equation (19) so that the next iteration $p^{\left(k+1\right)}$ is given by

(22) $$p^{\left(k+1\right)}=\underset{p}{\min\{}f(p^{\left(k\right)})+{\left(p-p^{\left(k\right)}\right)}^{H}\nabla f(p^{\left(k\right)})+\frac{\sum_{h=1}^{H}{\left\|p[h]-p^{\left(k\right)}[h]\right\|}_{2}^{2}}{2\text{Σ}}+v(p)\}$$

By further derivation, Equation (22) can be simplified to

(23) $$p^{\left(k+1\right)}=\underset{p}{\min\{}\sum_{h=1}^{H}[\frac{1}{2\text{Σ}}{\left\|p[h]-u^{\left(k\right)}[h]\right\|}_{2}^{2}+\lambda_{p}{\left\|p[h]\right\|}_{k}^{l}]\}$$

where $u^{\left(k\right)}[h]=p^{\left(k\right)}[h]-J^{\left(k\right)}[h]$. As one may note, the objective function in Equation (23) is separable, and thus *H* blocks of $p^{\left(k+1\right)}$ can be solved in a parallel manner. Although it is hard to solve *H* blocks, the classical BCD method has provided a critical inspiration, fortunately. The solution to the *h*th block of $p^{\left(k+1\right)}$ is given by a soft-thresholding operator [22]

(24) $$p^{\left(k+1\right)}[h]=\frac{u^{\left(k\right)}[h]}{{\left\|u^{\left(k\right)}[h]\right\|}_{2}}({\left\|u^{\left(k\right)}[h]\right\|}_{2}-{\text{λ}}_{p}{\left\|\Delta [h]\right\|}_{2})I({\left\|u^{\left(k\right)}[h]\right\|}_{2}\geq {\text{λ}}_{p}{\left\|\Delta [h]\right\|}_{2}),\;\;\;h=1,2,\cdots ,H$$

where I(⋅) denotes an indicator function. Instead of reconstructing $p^{\left(k+1\right)}$ directly, $p^{\left(k+1\right)}$ can be reconstructed by *H* blocks, which may be zero vectors during the iteration. It can be seen in Equation (24) that we just need to determine the relation of ${\left\|u^{\left(k\right)}[h]\right\|}_{2}$ and ${\text{λ}}_{p}{\left\|\Delta [h]\right\|}_{2}$ to judge whether $p^{\left(k+1\right)}[h]$ is a zero vector. If ${\left\|u^{\left(k\right)}[h]\right\|}_{2}$ is less than ${\text{λ}}_{p}{\left\|\Delta [h]\right\|}_{2}$, $p^{\left(k+1\right)}[h]$ must be a zero vector. Therefore, a considerable amount of computations can be avoided in the process of solving *H* blocks. Similarly, by utilizing the soft-thresholding operator, the solution to the *h*th block of $q^{\left(k+1\right)}$ is expressed as

(25) $$q^{\left(k+1\right)}[h]=\frac{{\bar{u}}^{\left(k\right)}[h]}{{\left\|{\bar{u}}^{\left(k\right)}[h]\right\|}_{2}}({\left\|{\bar{u}}^{\left(k\right)}[h]\right\|}_{2}-{\text{λ}}_{q}{\left\|\bar{\Delta }[h]\right\|}_{2})I({\left\|{\bar{u}}^{\left(k\right)}[h]\right\|}_{2}\geq {\text{λ}}_{q}{\left\|\bar{\Delta }[h]\right\|}_{2}),\;\;\;h=1,2,\cdots ,H$$

where $\bar{\Delta }[h]$ and ${\bar{J}}^{\left(k\right)}[h]$ are the *h*th blocks of $\bar{\Delta }$ and $\bar{J}$ with the length *d*, respectively, and ${\bar{u}}^{\left(k\right)}[h]=q^{k}[h]-{\bar{J}}^{\left(k\right)}[h]$. Following the similar derivation, we have

(26) $${\bar{J}}^{\left(k\right)}=diag(\bar{\Delta })D^{H}\cdot [\sum_{h=1}^{H}(C[h]p[h]+D[h]q^{\left(k\right)}[h])-z]$$

where $\bar{\Delta }={\left[{\bar{\Delta }}_{1},{\bar{\Delta }}_{2},\cdots ,{\bar{\Delta }}_{L}\right]}^{T}$ is a L×1 vector and ${\bar{\Delta }}_{i}=\frac{1}{{\left\|{\hat{b}}^{H}({\tilde{\theta }}_{l})⊗\hat{b}({\tilde{\theta }}_{l})\right\|}_{2}^{2}}$, l=1,2,⋯,L. Based on Equations (24) and (25), p and q can be reconstructed alternately until the following criterion is satisfied

(27) $$\left\|\frac{{\text{η}}^{\left(k+1\right)}-{\text{η}}^{\left(k\right)}}{{\text{η}}^{\left(k\right)}}\right\|\leq \text{γ}$$

where ${\text{η}}^{\left(k\right)}=[p^{\left(k\right)};q^{\left(k\right)}]$ and γ is the small tolerance.

To reduce the computational complexity, a block selection criterion is given. This criterion is of great importance in the whole ABCD method. By utilizing the block selection criterion, we can only update the block that is the closest to z, *i.e.*,

(28) $$h_{0}=\arg\min{\left\|w[h]-z\right\|}_{2},\;\;\;\;\;h=1,2,\cdots ,H$$

where w[h]=C[h]p[h]+D[h]q[h]. This means that $p^{\left(k+1\right)}[h_{0}]$ and $q^{\left(k+1\right)}[h_{0}]$ at the *k*th iteration are updated as Equations (24) and (25) while the remaining blocks keep unchanged. The purpose of utilizing this block selection criterion is to avoid the update of repetitive and unnecessary blocks and reduce the computational complexity. The major steps of reconstructing p and q by the ABCD method are given as follows:

Initialization: set $k=0,p^{\left(0\right)}=0,q^{\left(0\right)}=0,C\in {\mathbb{C}}^{N^{2}\times L},D\in {\mathbb{C}}^{N^{2}\times L},z=vec(R_{Y})\in {\mathbb{C}}^{N^{2}\times 1}$ and $\text{γ}=10^{-5}$.

(1) Calculate $J^{\left(k\right)}$ and ${\bar{J}}^{\left(k\right)}$ in terms of Equations (21) and (26).
(2) Due to *H* blocks of $J^{\left(k\right)}$ and ${\bar{J}}^{\left(k\right)}$, calculate $u^{\left(k\right)}[h]$ and ${\bar{u}}^{\left(k\right)}[h]$, h=1,2,⋯,H.
(3) Calculate $p^{\left(k+1\right)}[h]$ and $q^{\left(k+1\right)}[h]$, h=1,2,⋯,H, in terms of (24) and (25).
(4) Choose the block index $h_{0}$ according to (28). Then, $p^{\left(k+1\right)}$ and $q^{\left(k+1\right)}$ are respectively updated as ${\left[p^{\left(k\right)}[1],\cdots ,p^{\left(k\right)}[h_{0}-1],p^{\left(k+1\right)}[h_{0}],p^{\left(k\right)}[h_{0}+1],\cdots ;p^{\left(k\right)}[H]\right]}^{T}$ and $[q^{\left(k\right)}[1],\cdots ,q^{\left(k\right)}[h_{0}-1],$ $q^{\left(k+1\right)}[h_{0}],q^{\left(k\right)}[h_{0}+1],\cdots ,q^{\left(k\right)}[H]{]}^{T}$.
(5) If $\left\|\frac{{\text{η}}^{\left(k+1\right)}-{\text{η}}^{\left(k\right)}}{{\text{η}}^{\left(k\right)}}\right\|\leq \text{γ}$, stop the iteration. Otherwise, set k=k+1 and return to step (1).

## 4. Global Convergence of the ABCD Method

The global convergence of the ABCD method is proved in this section. By combining the existing convergence proof of the general BCD framework [23] with ABCD method, a detailed derivation process for proving the global convergence is shown as follows.

First, we introduce the general BCD framework. Note that f(p) in Equation (18) is a continuous convex function and v(p) in (18) is a non-smooth convex function. Given a fixed point $p^{\left(k\right)}$, F(p) in Equation (18) can be approximated as the following form by exploiting the second order Taylor expansion of f(p) in the general BCD framework.

(29) $$F(p)\approx f(p^{\left(k\right)})+{\left(p-p^{\left(k\right)}\right)}^{H}\nabla f(p^{\left(k\right)})+\frac{1}{2}{\left(p-p^{\left(k\right)}\right)}^{H}H(p-p^{\left(k\right)})+v(p)$$

where $H=\nabla f^{2}(p^{\left(k\right)})=C^{H}C$ is the L×L Hessian matrix of $f(p^{\left(k\right)})$ with respect to $p^{\left(k\right)}$. It is clear that minimizing Equation (29) involves finding the next iteration $p^{\left(k+1\right)}$. Assume that χ=[1,2,⋯,H] is an *H*-dimensional index set and ${\text{χ}}_{0}$ is a subset of χ consisting of at most *K* indexes obtained by Equation (28). Hence, the next iteration $p^{\left(k+1\right)}$ is represented as

(30) $$p^{\left(k+1\right)}=\Lambda (p^{\left(k\right)},{\text{χ}}_{0})=\arg\underset{p}{\min}\{F(p)/p^{\left(k\right)}[h]=0,h\in \text{χ},h\notin {\text{χ}}_{0}\}$$

Then, to prove the global convergence, we give the modified Armijo rule and modified Gauss-Southwell-*r* rule that are prerequisites to guarantee the global convergence. These two rules are described in the following.

(1) Modified Armijo rule: If $0<{\text{α}}^{\left(k\right)}<1$, 0<ξ<1 and 0<κ<1, the following inequality holds:
  (31) $$F(p^{\left(k\right)}+{\text{α}}^{\left(k\right)}{\text{μ}}^{\left(k\right)})\leq F(p^{\left(k\right)})+{\text{α}}^{\left(k\right)}\text{κ}\Gamma^{\left(k\right)}$$
  where ${\text{μ}}^{\left(k\right)}=p-p^{\left(k\right)}$ and $\Gamma^{\left(k\right)}={\left({\text{μ}}^{\left(k\right)}\right)}^{H}\nabla f(p^{\left(k\right)})+\text{ξ}{\left({\text{μ}}^{\left(k\right)}\right)}^{H}H{\text{μ}}^{\left(k\right)}+v(p)-v(p^{\left(k\right)})$.
(2) Modified Gauss-Southwell-*r* rule: In the iteration, the index set ${\text{χ}}_{0}$ obtained by Equation (28) must satisfy
  (32) $${\left\|\Lambda (p^{\left(k\right)},\chi_{0})\right\|}_{2}\leq \text{ς}{\left\|\Lambda (p^{\left(k\right)},\text{χ})\right\|}_{2}$$
  where 0<ς<1.

It is well known that the problem of proving the global convergence is quite complex and intractable. However, fortunately, since Equations (16) and (17) are both convex and have both only one global point, we only need to show that Equations (16) and (17) satisfy the modified Armijo rule and modified Gauss-Southwell-*r* rule to prove the global convergence of the ABCD method. Furthermore, since Equation (16) has the same structure as Equation (17), it is enough to just prove that Equation (16) satisfies the modified Armijo rule and modified Gauss-Southwell-*r* rule. Regarding Equation (17), the derivation process is given in the same way.

To see the first, the following inequality holds according to Equation (30).

(33) $$F(p)=F(p^{\left(k\right)}+{\text{μ}}^{\left(k\right)})\leq F(p^{\left(k\right)}+{\text{α}}^{\left(k\right)}{\text{μ}}^{\left(k\right)})$$

By substituting Equation (33) into Equation (29), we have

(34) $$\begin{array}{l} f(p^{\left(k\right)})+{\left({\text{μ}}^{\left(k\right)}\right)}^{H}\nabla f(p^{\left(k\right)})+\frac{1}{2}{\left({\text{μ}}^{\left(k\right)}\right)}^{H}H({\text{μ}}^{\left(k\right)})+v(p^{\left(k\right)}+{\text{μ}}^{\left(k\right)})\leq \\ f(p^{\left(k\right)})+{\left({\text{α}}^{\left(k\right)}{\text{μ}}^{\left(k\right)}\right)}^{H}\nabla f(p^{\left(k\right)})+\frac{1}{2}{\left({\text{α}}^{\left(k\right)}{\text{μ}}^{\left(k\right)}\right)}^{H}H({\text{α}}^{\left(k\right)}{\text{μ}}^{\left(k\right)})+v(p^{\left(k\right)}+{\text{α}}^{\left(k\right)}{\text{μ}}^{\left(k\right)}) \end{array}$$

Based on the fact that $v(p^{\left(k\right)}+{\text{α}}^{\left(k\right)}{\text{μ}}^{\left(k\right)})\leq {\text{α}}^{\left(k\right)}v(p^{\left(k\right)}+{\text{μ}}^{\left(k\right)})+(1-{\text{α}}^{\left(k\right)})v(p^{\left(k\right)})$, Equation (34) can be further simplified to

(35) $$(1-{\text{α}}^{\left(k\right)}){\left({\text{μ}}^{\left(k\right)}\right)}^{H}\nabla f(p^{\left(k\right)})+\frac{1}{2}[1-{\left({\text{α}}^{\left(k\right)}\right)}^{2}]{\left({\text{μ}}^{\left(k\right)}\right)}^{H}H({\text{μ}}^{\left(k\right)})+(1-{\text{α}}^{\left(k\right)})[v(p^{\left(k\right)}+{\text{μ}}^{\left(k\right)})-v(p^{\left(k\right)})]\leq 0$$

Since $0<{\text{α}}^{\left(k\right)}<1$, by dividing by $1-{\text{α}}^{\left(k\right)}$ and setting ${\text{α}}^{\left(k\right)}\to 1$, we have

(36) $${\left({\text{μ}}^{\left(k\right)}\right)}^{H}\nabla f(p^{\left(k\right)})+{\left({\text{μ}}^{\left(k\right)}\right)}^{H}H({\text{μ}}^{\left(k\right)})+v(p^{\left(k\right)}+{\text{μ}}^{\left(k\right)})-v(p^{\left(k\right)})\leq 0$$

For 0<ξ<1, it can be deduced from (36) that

(37) $$\Gamma^{\left(k\right)}={\left({\text{μ}}^{\left(k\right)}\right)}^{H}\nabla f(p^{\left(k\right)})+\text{ξ}{\left({\text{μ}}^{\left(k\right)}\right)}^{H}H{\text{μ}}^{\left(k\right)}+v(p)-v(p^{\left(k\right)})\leq (\text{ξ}-1){\left({\text{μ}}^{\left(k\right)}\right)}^{H}H{\text{μ}}^{\left(k\right)}\leq 0$$

Subsequently, by exploiting the convexity of f(p), we obtain

(38) $$f(p)=f(p^{\left(k\right)}+{\text{μ}}^{\left(k\right)})\leq f(p^{\left(k\right)})+{\left({\text{μ}}^{\left(k\right)}\right)}^{H}\nabla f(p^{\left(k\right)})+\frac{1}{2}{\left({\text{μ}}^{\left(k\right)}\right)}^{H}H({\text{μ}}^{\left(k\right)})$$

Since F(p)=f(p)+v(p), it is nature to have

(39) $$F(p)=F(p^{\left(k\right)}+{\text{μ}}^{\left(k\right)})\leq F(p^{\left(k\right)})+{\left({\text{μ}}^{\left(k\right)}\right)}^{H}\nabla f(p^{\left(k\right)})+\frac{1}{2}{\left({\text{μ}}^{\left(k\right)}\right)}^{H}H({\text{μ}}^{\left(k\right)})+v(p)-v(p^{\left(k\right)})$$

Following the fact in Equation (37), the following inequality holds

(40) $$\begin{array}{l} F(p^{\left(k\right)})+{\left({\text{μ}}^{\left(k\right)}\right)}^{H}\nabla f(p^{\left(k\right)})+\frac{1}{2}{\left({\text{μ}}^{\left(k\right)}\right)}^{H}H({\text{μ}}^{\left(k\right)})+v(p)-v(p^{\left(k\right)})\\ \leq F(p^{\left(k\right)})+\text{κ}[{\left({\text{μ}}^{\left(k\right)}\right)}^{H}\nabla f(p^{\left(k\right)})+\frac{1}{2}{\left({\text{μ}}^{\left(k\right)}\right)}^{H}H({\text{μ}}^{\left(k\right)})+v(p)-v(p^{\left(k\right)})] \end{array}$$

where 0<κ<1. Combining Equations (39) and (40), we have

(41) $$F(p^{\left(k\right)}+{\text{μ}}^{\left(k\right)})\leq F(p^{\left(k\right)})+\text{κ}[{\left({\text{μ}}^{\left(k\right)}\right)}^{H}\nabla f(p^{\left(k\right)})+\frac{1}{2}{\left({\text{μ}}^{\left(k\right)}\right)}^{H}H({\text{μ}}^{\left(k\right)})+v(p)-v(p^{\left(k\right)})]$$

It is worth pointing out that Equation (41) is equal to Equation (31) with ${\text{α}}^{\left(k\right)}=1$ and $\text{ξ}=\frac{1}{2}$. Thus, it has been proved that Equation (16) satisfies the modified Armijo rule.

Secondly, in order to prove that Equation (16) satisfies the modified Gauss-Southwell-*r* rule, the following form is shown

(42) $$h_{0}=\arg\min{\|w[h]-z\|}_{2}=\arg\min{\left\|\Lambda (p^{\left(k\right)},{\text{χ}}_{i})\right\|}_{2}$$

where the cardinality of ${\text{χ}}_{i}$ is the same as that of ${\text{χ}}_{0}$. Without loss of generality, consider the worst case, *i.e.*, the cardinality of ${\text{χ}}_{i}$ is the maximization *K*, and assume H=KZ so that χ is expressed as $\text{χ}=[{\text{χ}}_{1}，{\text{χ}}_{2}，\cdots ，{\text{χ}}_{Z}]$. Thus, we have

(43) $${\left\|\Lambda (p^{\left(k\right)},{\text{χ}}_{0})\right\|}_{2}\leq {\left\|\Lambda (p^{\left(k\right)},{\text{χ}}_{i})\right\|}_{2}$$

Based on the following equation

(44) $${\left\|\Lambda (p^{\left(k\right)},\text{χ})\right\|}_{2}=\sqrt{\sum_{z=1}^{Z}{\left\|\Lambda (p^{\left(k\right)},{\text{χ}}_{z})\right\|}_{2}^{2}}$$

it is easy to have

(45) $${\left\|\Lambda (p^{\left(k\right)},{\text{χ}}_{0})\right\|}_{2}\leq \frac{1}{\sqrt{Z}}{\left\|\Lambda (p^{\left(k\right)},\text{χ})\right\|}_{2}$$

Since Equation (45) is equal to Equation (32) with $\varsigma =\frac{1}{\sqrt{Z}}$, Equation (16) satisfies the modified Gauss-Southwell-*r* rule. Therefore, based on the above analysis, the global convergence of the ABCD method has been proved.

## 5. Simulation Results

This section presents several simulations to validate the superior performance of the proposed method as compared with R-GBCD+ and OGSBI-SVD. The angular space [−90°, 90°] is taken the grid with grid interval τ = 3° to perform three methods for off-grid targets. We set the length of block, the number of ULA sensors and spacing between adjacent sensors to be d=3, M=9 and $\overset{⌢}{d}=\frac{\text{λ}}{2}$, respectively. In the simulation, the root mean squared error (RMSE) and success rate of DOA estimation are two significant performance indexes. RMSE is defined as

(46) $$RMSE=\sqrt{\sum_{i=1}^{Mc}\sum_{k=1}^{K}\frac{{\left({\bar{\theta }}_{k,i}-\theta_{k}\right)}^{2}}{K\cdot Mc}}$$

where Mc is the number of Monte Carlo runs and ${\bar{\theta }}_{k,i}$ is the estimate of $\theta_{k}$ in the *i*th Monte Carlo run, and success rate is declared if the estimation error is within a certain small Euclidean distance of the true directions.

**Figure 1:**
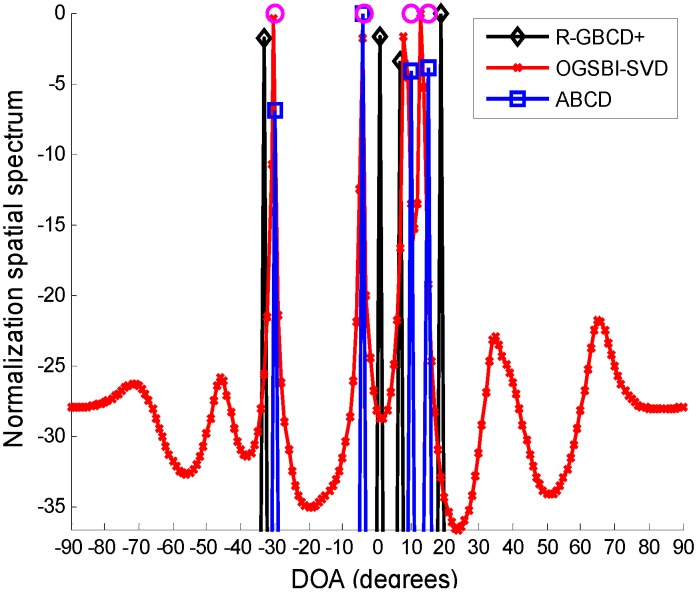
Spatial spectra of R-GBCD+, OGSBI-SVD and ABCD, where the pink circles denote the true DOAs.

In the first simulation, we compare the spatial spectra of R-GBCD+, OGSBI-SVD and ABCD. Consider four far-field narrowband sources impinging on the ULA from [−30.4° −3.8° 10.1° 15.3°], where the latter two most closely spaced sources are coherent and the remaining sources are independent of other sources. Figure 1 presents the spatial spectra of R-GBCD+, OGSBI-SVD and ABCD with SNR 3 dB and number of snapshots 100. For the convenience of analysis, the spatial spectra are normalized. We can see from Figure 1 that the spatial spectra of three methods are able to detect four sources, but the spatial spectrum obtained by R-GBCD+ has obvious bias at the true directions and OGSBI-SVD can yield slight bias in the vicinity of the coherent sources. Note that ABCD has a nearly ideal spatial spectrum, and thus it outperforms R-GBCD+ and OGSBI-SVD in terms of the spatial spectrum.

**Figure 2:**
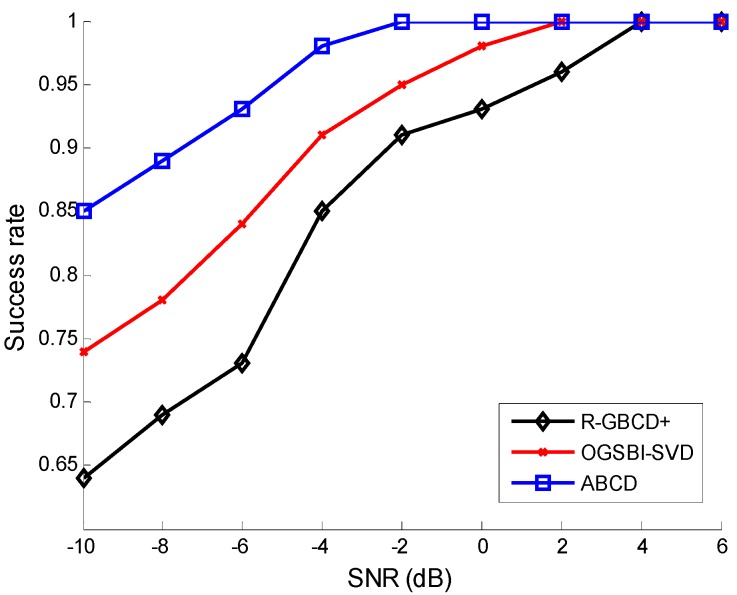
Success rates *vs.* SNR with the fixed number of snapshots 100.

**Figure 3:**
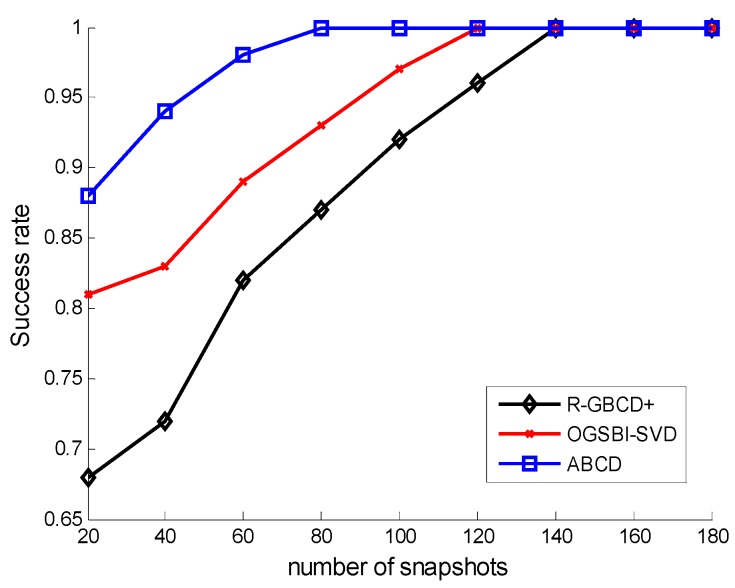
Success rates *vs.* number of snapshots with the fixed SNR 0 dB.

The success rates of three methods *vs.* SNR and the number of snapshots are analyzed in the second simulation. The source mode is the same as the first simulation. Figure 2 shows the success rates of three methods *vs.* SNR with the fixed number of snapshots 100, whereas the success rates of three methods *vs.* number of snapshots are depicted with the fixed SNR 0 dB in Figure 3. The following facts can be acquired from Figure 2 and Figure 3 that three methods can estimate correctly for high SNR or large number of snapshots and ABCD has a higher success rate than the other two methods for low SNR or a small number of snapshots.

**Figure 4:**
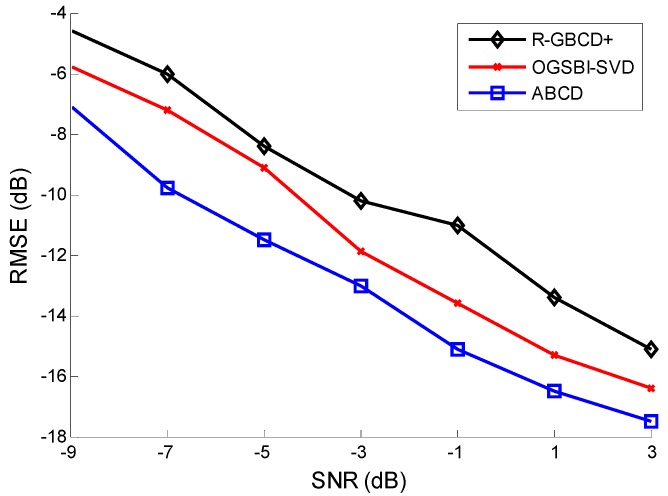
RMSE *vs.* SNR with the fixed number of snapshots 100.

**Figure 5:**
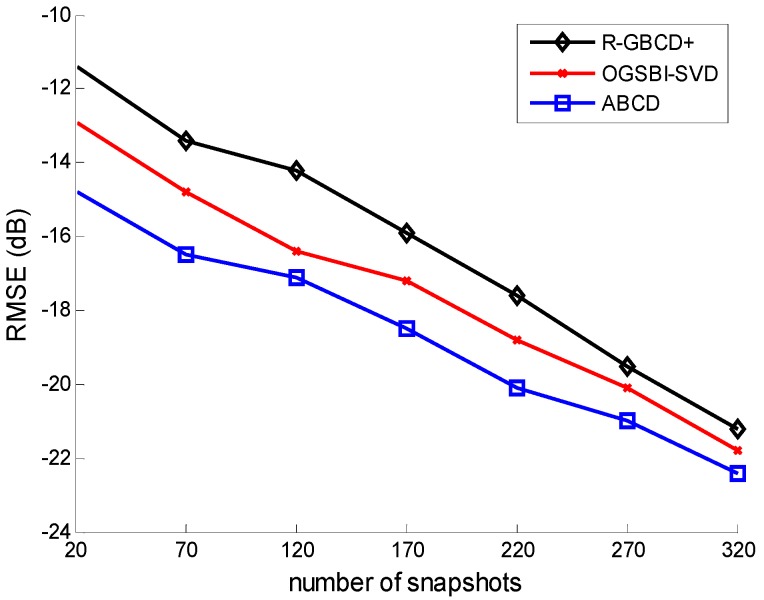
RMSE *vs.* number of snapshots with the fixed SNR 0 dB.

The third simulation considers the RMSE of three methods *vs.* SNR and the number of snapshots. All the conditions are the same as the second simulation. Figure 4 and Figure 5 show the RMSE of three methods *vs.* SNR and the number of snapshots, respectively. It is indicated in Figure 4 and Figure 5 that ABCD has the best estimation accuracy among all three methods. Moreover, the accuracy of three methods is gradually improving with SNR or the number of snapshots increasing.

**Figure 6:**
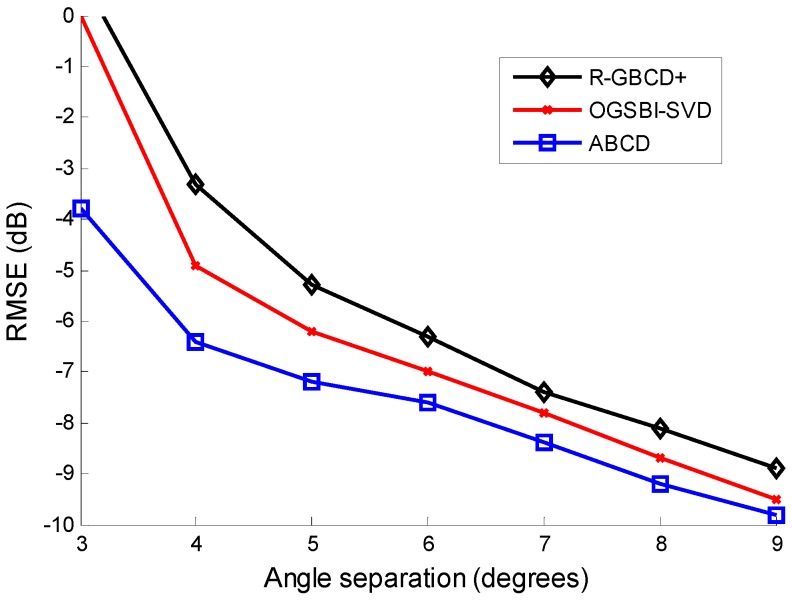
RMSE *vs.* angle separation with the fixed SNR 0 dB and number of snapshots 100 for coherent sources.

Finally, we test the resolving ability by showing the relation between RMSE of three methods and angle separation of sources, which is illustrated in Figure 6 Consider two coherent sources impinging on the ULA from 30.7 and 30.7+Δθ, where the step of Δθ is 1°. The SNR is 0 dB and the number of snapshots is 100. As can be seen from Figure 6, the performance of R-GBCD+ and OGSBI-SVD degrades severely as angle separation is 3°, while ABCD can still provide a precise estimation as long as angle separation is no less than 3°. The proposed ABCD is the most accurate method and has higher resolution than the other two methods.

## 6. Conclusions

In this paper, a novel ABCD method is proposed for off-grid DOA estimation in CS. The proposed method minimizes the mixed *k*-*l* norm to reconstruct the sparse source and estimate the grid error. In order to make the minimization problem tractable, an iterative process that minimizes the mixed *k*-*l* norm alternately over two sparse vectors is adopted. By reconstructing the block sparse source instead of conventional sparse source, the proposed method can achieve the better reconstruction properties. A block selection criterion is given to update the block so that the proposed method can reduce computational complexity. It is proved that the proposed method has the global convergence. Simulation results show that the proposed method has more notable performance advantages than R-GBCD+ and OGSBI-SVD in terms of spatial spectrum, RMSE and success rate.
